# Medicinal Cannabis in California: An Interview with Igor Grant, MD

**DOI:** 10.1089/can.2016.29005.igr

**Published:** 2016-02-01

**Authors:** Daniele Piomelli, Igor Grant

**Affiliations:** ^1^ Department of Anatomy and Neurobiology, University of California Irvine, Irvine, California.; ^2^ Department of Drug Discovery and Development, Istituto Italiano di Tecnologia, Genoa, Italy.; ^3^ Department of Psychiatry, Center for Medicinal Cannabis Research, University of California San Diego, San Diego, California.

**Keywords:** medical Cannabis, multiple sclerosis, chronic pain, HIV, marijuana

## Abstract

Dr. Igor Grant, MD, is distinguished professor and chair of psychiatry and director of the HIV Neurobehavioral Research Program and the Center for Medicinal Cannabis Research at the University of California, San Diego. Dr. Grant is a neuropsychiatrist who graduated from the University of British Columbia School of Medicine (1966), and received specialty training in psychiatry at the University of Pennsylvania (1967–1971), and additional training in neurology at the Institute of Neurology (1980–1981), London, U.K. Dr. Grant's academic interests focus on the effects of various diseases on brain and behavior, with an emphasis on translational studies in HIV, and drugs of abuse. He has contributed to ∼700 scholarly publications and is principal investigator of several NIH studies, including an NIDA P50 (Translational Methamphetamine AIDS Research Center—TMARC), and is codirector of the HIV Neurobehavioral Research Center (HNRC).

***Cannabis and Cannabinoid Research* (Dr. Daniele Piomelli: *CCR*): The Center for Medicinal Cannabis Research (CMCR) was created to provide evidence-based answers to the question “Does marijuana have therapeutic value”? What was the legislative backdrop to its creation?**

**Dr. Grant:** The CMCR was established after the passage of Proposition 215, The Compassionate Use Act, in 1996. That legislation envisioned permitting medical Cannabis to patients whose conditions warranted it. Senator John Vasconcellos of the California Senate wished to establish a scientific basis for medical Cannabis recommendations, and in what specific conditions that it might be useful. Working with the University of California, he authored legislation to enable the establishment of CMCR. The purpose of the program was to conduct clinical trials on medical Cannabis, as well as perform a limited number of pre-clinical studies.

***CCR*: For how long did the State of California fund research at the CMCR?**

**Dr. Grant:** Funding for the CMCR began in 2001. The funding was allocated in three successive years. However, CMCR was allowed to retain carryforward funds to complete the various studies. Because regulatory and other issues caused some delays in the initial implementation of the studies, and also because the actual conduct of the studies took some period of time, the final study that CMCR supported was not actually completed until 2014.

***CCR*: CMCR supported both clinical and pre-clinical studies, after rigorous peer reviewing. Were most of the studies focused on one therapeutic area?**

**Dr. Grant:** Neuropathic pain and spasticity related to multiple sclerosis.

***CCR*: Can you single out one clinical study (or series of studies) funded by the CMCR that you consider particularly significant?**

**Dr. Grant:** I am not able to single out a particular study. I think that, as a body of work, what the CMCR was able to establish was that it is possible to do studies with inhaled Cannabis in a manner that was safe, tolerable by patients, and which did not result in any serious side effects. In addition, people with neuropathic pain experienced clear benefit in the short term and this benefit was over and above their usual neuropathic pain treatments. The short-term effectiveness of Cannabis was comparable to that of currently existing treatments.

***CCR*: What studies do you think should have been supported?**

**Dr. Grant:** I would like to see supported much more extensive and representative clinical trials in medicinal Cannabis. The studies CMCR conducted were brief in nature. While I am confident that our results will be replicated, that is, others will also conclude that in the short-term Cannabis is useful in the treatment of neuropathic pain and spasticity in multiple sclerosis, which were the two conditions we studied. We do not know what the long-term effectiveness of Cannabis may be. For example, is it possible that after 6 months, or a year, these beneficial effects disappear? I have said that the side effects were mild and not worrisome; however, this was in the short term. Is it possible that more serious side effects could develop after long-term exposure? We also do not know whether inhaled Cannabis actually works more reliably than orally administered Cannabis. Certainly there has been speculation to this effect based on the irregular absorption of cannabinoids from the gut, and the effects of first pass metabolism that may result in unpredictable levels of THC in the blood.

To address these points, it would be useful to conduct larger scale studies with people who may have different medical conditions, like neuropathic pain in older people who may have mild heart disease or diabetes, to determine safety in such populations. If studies were extended, we could better answer questions about longer term effectiveness and possible toxicity. By conducting studies that involved different modes of administration, we could answer questions about whether inhalation is truly superior. In addition, by having treatment arms that involve pure THC administered by mouth or sublingually and/or administering synthetic CB1 and CB2 agonists, partial agonists, and fatty acid amide hydrolase (FAAH) inhibitors, we may be able to answer questions regarding the usefulness of such cannabinoid modulators that might make it less necessary to use inhaled Cannabis.

***CCR*: What difficulties did CMCR encounter in accomplishing its mission?**

**Dr. Grant:** We did not encounter any serious difficulties in the conduct of our studies, other than that navigating the regulatory waters was complicated. CMCR needed to get approval from State regulators (Research Advisory Panel of California), as well as Federal regulators and approvers (Department of Health and Human Services; NIDA; DEA; FDA). These processes all took time, especially initially because several Federal agencies had not actually worked together on approving such clinical trials in the recent past. Even when the process was working smoothly, however, the fastest approvals would have been in just more than 6 months and the average time was 1 year. Recent changes promulgated by the Obama administration do cut out the U.S. Department of Health and Human Services review level, so this should streamline Federal approval. Overall, there were no particular hurdles.

***CCR*: Any final comments?**

**Dr. Grant:** My final comments would be that it would be really useful to have much more extended clinical trials on Cannabis and its constituents, be they natural or synthetic, conducted over longer periods of time and applied to conditions where there is at least anecdotal suggestion of benefit. We have already found evidence that Cannabis is probably useful in the treatment of neuropathic pain and spasticity of multiple sclerosis, and other data have shown value in the treatment of nausea and improving weight gain. There are intriguing suggestions of unique value of cannabidiol in the treatment of certain forms of epilepsy that are resistant to other treatments. Other clues are that the cannabinoids, particularly cannabidiol, may have value as anti-inflammatory agents in conditions such as inflammatory bowel disease, and possibly anxiety and even treatment of psychosis. We do need clinical trials in these areas. I do think it is entirely possible that modulators of the endocannabinoid system, including drugs such as inhibitors of endocannabinoid hydrolysis, FAAH inhibitors, will be valuable new tools in neuropsychiatry and medicine.

## Abbreviations Used

CCR: Cannabis and Cannabinoid Research
CMCR: Center for Medicinal Cannabis Research
FAAH: fatty acid amide hydrolase
THC: tetrahydrocannabinol

